# Pine as Fast Food: Foraging Ecology of an Endangered Cockatoo in a Forestry Landscape

**DOI:** 10.1371/journal.pone.0061145

**Published:** 2013-04-11

**Authors:** William D. Stock, Hugh Finn, Jackson Parker, Ken Dods

**Affiliations:** 1 Centre for Ecosystem Management, Edith Cowan University, Joondalup, Western Australia, Australia; 2 School of Biological Sciences and Biotechnology, Murdoch University, Perth, Western Australia, Australia; 3 Department of Agriculture and Food, Western Australia, South Perth, Western Australia, Australia; 4 ChemCentre, Bentley, Western Australia, Australia; Liverpool John Moores University, United Kingdom

## Abstract

Pine plantations near Perth, Western Australia have provided an important food source for endangered Carnaby’s Cockatoos (*Calyptorhynchus latirostris*) since the 1940s. Plans to harvest these plantations without re-planting will remove this food source by 2031 or earlier. To assess the impact of pine removal, we studied the ecological association between Carnaby’s Cockatoos and pine using behavioural, nutritional, and phenological data. Pine plantations provided high densities of seed (158 025 seeds ha^−1^) over a large area (c. 15 000 ha). Carnaby’s Cockatoos fed throughout these plantations and removed almost the entire annual crop of pine cones. Peak cockatoo abundance coincided with pine seed maturation. Pine seed had energy and protein contents equivalent to native food sources and, critically, is available in summer when breeding pairs have young offspring to feed. This strong and enduring ecological association clearly suggests that removing pine will have a significant impact on this endangered species unless restoration strategies, to establish alternative food sources, are implemented.

## Introduction

Carnaby’s Cockatoo (*Calyptorhynchus latirostris*) is a threatened avifauna endemic to southwestern Australia that has declined by more than 50% over the last half-century [1], [2]. The species is listed as endangered under the Commonwealth *Environment Protection and Biodiversity Conservation Act 1999* [3] and as a Schedule 1 species (‘Fauna that is rare or likely to become extinct’) under the Western Australian *Wildlife Conservation Act 1950* [4], and is classified as Endangered under the IUCN Red List of Threatened Species [5]. Carnaby’s Cockatoos are vulnerable to the continued loss of breeding and feeding habitat from land-clearing [2], [6], [7], [8]. Here we describe the ecological association between Carnaby’s Cockatoos and an abundant and valuable anthropogenic food source–plantation pine–on the Swan Coastal Plain north of Perth, Western Australia.

Carnaby’s Cockatoos are called the ‘rain bird’ because they move into areas of high rainfall along the coast after breeding in drier areas inland [9], [10]. This seasonal shift brings between 4 600 to 15 000 out onto the northern Swan Coastal Plain along the western coast of Western Australia from about January–June, meaning that the region supports the largest population of non-breeding birds in southwestern Australia [6]. Carnaby’s Cockatoos feed on a range of foods on the Swan Coastal Plain, including seeds and flowers of proteaceous shrubs and myrtaceous trees within remnant heath and woodland habitats, as well seeds of plantation pine (*Pinus* spp.) [8], [9], [10], [11], [12], [13], [14], [15]. When they arrive on the Swan Coastal Plain, Carnaby’s Cockatoos encounter an landscape that, while vegetatively distinct from the sandplain heath (kwongan) and eucalypt woodlands occurring inland, is almost equally modified by land-clearing [16], [17]. Urban development in Australia continues to rely on the clearing of bushland, leading to small but cumulative losses of feeding habitat for native fauna [18]. In the Perth metropolitan region, for example, 5974 ha of native vegetation were cleared between 1998 and 2004 [19]. These losses add to historical losses for agriculture, industry, and housing, with the Swan Coastal Plain around Perth retaining less than 30% of its original (pre-European settlement) native vegetation cover [19]. Remaining native bushland is also under pressure from regular arson fires, plant pathogens such as *Phytopthora cinnamomi* (dieback), climate change, and other stressors [20], [21]. These factors make the loss of feeding habitat on the Swan Coastal Plain an important conservation concern for Carnaby’s Cockatoos and emphasise the need to conserve natural and anthropogenic food sources wherever possible [6], [7], [8].

Pine plantations have been an important food source for Carnaby’s Cockatoos on the Swan Coastal Plain for more than 75 years and flocks of up to several hundred birds have been commonly sighted within these plantations [8], [10], [12], [14]. The Gnangara pine (mostly Maritime Pine *Pinus pinaster*) plantations north of Perth are the largest pine plantation in the Perth region and, at their historical maximum in 2002, covered an area of 23 000 ha [22]. Since 2004, the Gnangara plantations have been harvested without replacement, which will result in the complete removal of all remaining stands of pine by 2031 or earlier [6], [22]. A regional planning process, known as the Gnangara Sustainability Strategy (GSS), proposed revegetating portions of the plantation system with native vegetation [22] but the State Government has yet to implement any revegetation activities more than three years after the draft strategy was released.

The lack of action to address the potential impacts of pine removal is concerning for three reasons. Firstly, the current Carnaby’s Cockatoo Recovery Plan states that failing to provide an alternative food source to pine is ‘likely to have a significant impact on the food resources available’ in the Perth region [6]. The plan also recognises that that there are significant economic costs associated with avoiding (e.g. retaining some pine) or mitigating (e.g. revegetating with native flora) this impact. This suggests that cost-benefit analyses will drive decision-making about avoidance and mitigation measures, emphasising the need to adequately characterise the food resource provided by the Gnangara plantation system. Secondly, the time lags and uncertainties associated with revegetation suggest that prompt initiation of revegetation is needed to avoid gaps between when pine is removed and when seeds and flowers from revegetation mature [23], [24]. Finally, although extensive areas of *Banksia* woodland remain on the northern Swan Coastal Plain, it remains to be shown whether food sources in this habitat (e.g. seeds from *Banksia* spp.) would be available across the entirety of the non-breeding period and therefore adequately replace pine seeds. These factors emphasise the need to determine the ecological value of pine to Carnaby’s Cockatoos, so that that impacts from pine removal can be properly assessed and mitigation and offset measures appropriately calculated. However, despite the historical association between pine plantations and Carnaby’s Cockatoos, little is known about the foraging ecology of the species within plantation landscapes and on the Swan Coastal Plain generally [7], [8], [10], [12].

To improve the scientific basis for decision-making, we examined the ecological association between Carnaby’s Cockatoos and the Gnangara pine plantation system north of Perth. We investigated three facets of the foraging ecology of Carnaby’s Cockatoos associated with the pine plantations. Firstly, we assessed spatial and temporal patterns in feeding activity and abundance by: (a) undertaking monthly counts of pine feeding residues at fixed sites; (b) counting feeding residues and standing crops of pine cones across the spatial and age range of pine plantations; and (c) conducting weekly roost counts at known over-night roost sites within or near to the Gnangara plantation system. Secondly, we examined the feeding behaviour of Carnaby’s Cockatoos associated with the Gnangara plantation system during the non-breeding period. We conducted behavioural observations of flocks within plantations and surrounding landscapes to describe: (a) the use of pine and other habitats for feeding and roosting; (b) food plant use and (c) daily activity patterns and time budgets. Finally, we assessed the characteristics of pine as a food source by: (a) monitoring the developmental status of pine cones over one year to assess seasonal patterns of cone maturation and seed availability and (b) conducted chemical analyses of pine seeds and native food plants to compare their relative energetic and nutritional qualities.

## Methods

### Ethics statement

Data collected adhered to the Australian legal requirements for the use of animals in research and was conducted with the approval of the Animal Ethics Committees of Edith Cowan University (Project 2675, Roost Monitoring) and Murdoch University (Project NS2239/09, Flock Follows). Field observations of Carnaby’s Cockatoos was conducted under a scientific purposes licence issued (to HF) by the WA Department of Environment and Conservation under the provisions of The Wildlife Conservation Act 1950 (WA). No specific permits were required for the other field studies of the pine because it is an introduced species. Permission to collect pine cones was given by the Forest Products Commission.

### Study area

**Figure 1:**
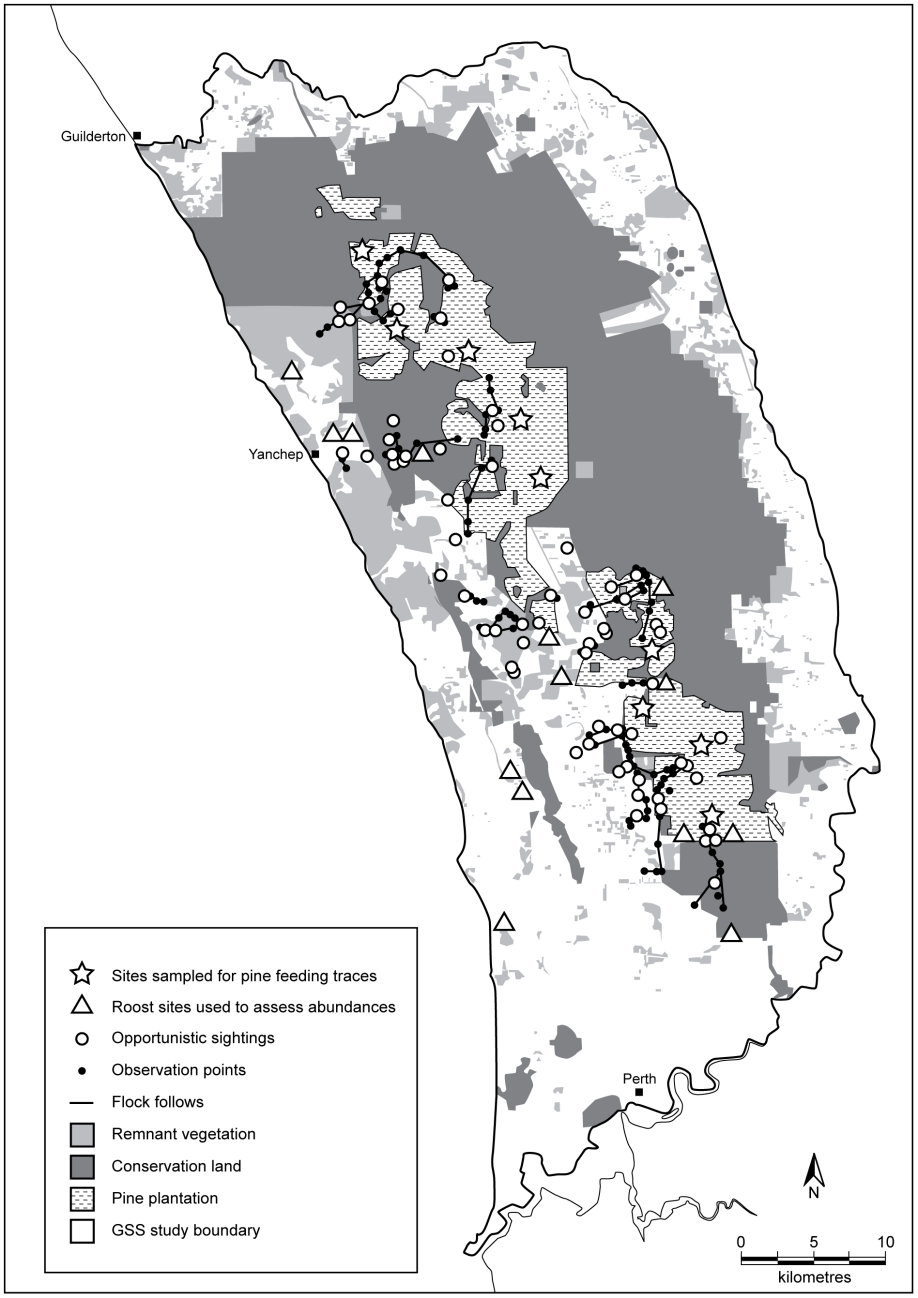
Locations of sightings, flock follows and point samples for flock follows. Map also shows the 9 sites which were sampled monthly to assess pine feeding traces and the 14 roost sites used to assess Carnaby’s Cockatoo abundances.

The study was conducted in the 2200 km^2^ area identified by the Gnangara Coordinating Committee as the focus of Gnangara Sustainability Strategy [22]. This area, which is approximately the spatial extent of the Gnangara superficial aquifer in the northern portions of Perth metropolitan area in Western Australia (Figure 1) includes three pine plantation systems (Gnangara, Pinjar, and Yanchep) that, at their maximum extent in 2002, covered an area of 23 000. The standing area of pine was c. 15 000 ha in 2009/2010 [8]. Remnant native vegetation also occurred within and adjacent to plantation stands (100 000 ha), and included areas of *Banksia* woodland (typically a mix of *Banksia attenuata and B*. *menziesii*), tuart (*Eucalyptus gomphocephala*) woodland, and thickets of the shrub *B. sessilis*. Currently there are no restoration efforts directed towards the return of native vegetation and natural regeneration of the area is too slow to offer any significant food sources for Carnaby’s Cockatoos in the foreseeable future.

### Pine availability, energetic and nutritional qualities

To determine when pine cones reached maturity, we collected cones from five branches of ten randomly selected pines (*Pinus pinaster*) felled during forestry operations each month from July 2009 to June 2010 and assessed the developmental stage of the cones. The cones were classified into age cohorts based on their position on the branch, dried (at 60°C), and weighed (dry mass). The age cohorts were decided from the position of cones located near the tips of the branches which were pollinated in that season versus those further down the branch which were pollinated in the previous season. All large cones were dried separately in paper bags and the total number of seeds per cone was determined. As *Pinus* spp. have a prolonged seed development, trees can contain cones of at least two ages since pollination.

To characterise the energetic and nutritional qualities of pine seeds relative to other food sources, we purchased seeds of 10 native and 2 non-native species from the Forest Products Commission. Those unavailable from this source (n = 4) were collected from a minimum of 5 parent plants occurring in the study area. A random subsample of 20 seeds was used to determine the average seed masses of each species as well as the ratio of cotyledon mass to total seed mass. To obtain a good estimate of the average nutritional composition of the seeds of each species a 5 g sample of seed was manually dehusked and the cotyledon material analysed for protein, fat and neutral detergent fibre [25]. Crude protein content was calculated from the nitrogen content measured on a Leco FP528 (St Joseph, Michigan) analysis using a conversion factor of 5.30 (N×5.30 conversion factor for nuts) [26] and fat contents were determined by solvent extraction using the Soxhlet method [27]. Four species with very small seeds were analysed as whole seeds. The energy content of each seed was then calculated from the proportional content of each component using the following conversion factors (1 g fat = 37 kJg^−1^, 1 g protein = 17 kJg^−1^ and 1 g carbohydrate = 16 kJg^−1^) [26]. The carbohydrate content of the seeds was determined by the difference method [28]. The energy contents of each plant species were then used to estimate the number of seeds required by Cockatoos to meet their field metabolic energetic requirements of 726 kJ d^−1^, as estimated by Cooper *et al*. [29].

### Carnaby’s Cockatoo abundances

To determine trends in the abundance of Carnaby’s Cockatoos using the Gnangara plantations, we counted the number of birds occupying known over-night roost sites [7], [30]. From February to September 2009, observers conducted simultaneous roost counts once a week at 14 known roost sites located in or around the plantations (Figure 1). We conducted the counts from February to September because previous studies had suggested that these months encompassed the period in which pine is most intensively used [7], [10], [14]. The counts were undertaken over a 1 hr period which included 30 mins before and after sunset. We determined monthly mean abundances from the weekly counts for the number birds present at roost sites (mean of weekly counts ± SE).

### Feeding activity within pine plantations

To quantify the spatial extent of feeding activity within the Gnangara plantations, we counted feeding traces (i.e. fallen pine cones with evidence of feeding by Carnaby’s Cockatoos) in 122 sampling plots (20 m×20 m) in July and August 2009. Carnaby’s Cockatoos are the only species that feeds on pine cones in the Gnangara plantations and the damage caused by feeding (e.g. bracts that are bent, shredded, or broken) is obvious, making feeding traces a reliable indicator of cockatoo feeding. The plots were distributed across 61 sites and were positioned throughout the Gnangara plantations so as to cover the full spatial extent of the plantations. Plots sampled the full range of stand ages, ranging from very young (16 years post-establishment) to very mature (78 years post-establishment). Cones on the ground were classified as either (1) fully/mostly eaten or (2) handled/partially eaten (<5% of the cone damaged). Carnaby’s Cockatoos often drop cones without fully consuming all seeds [10]. However, we did observe birds returning to consume seeds from previously dropped cones. We also estimated the number of cones remaining in the canopies of all the trees by scanning all branches of trees in the sample plots with binoculars.

To quantify temporal trends in feeding activity, we conducted transect sampling at nine sites across the Gnangara plantations (Figure 1). Sites ranged in age from 26 to 58 years old. At each site three permanent transects (100 m long×2 m wide) were established in July 2009. Transects were cleared of existing feeding traces and new feeding traces were counted each month for the next 11 months. After counting each month all transects were raked to remove feeding traces.

### Behavioural observations

We used behavioural observations to obtain information on the food plant use, use of habitats for feeding and roosting, and activity patterns of Carnaby’s Cockatoos associated with the pine plantations during the non-breeding period. Behavioural observations were conducted of flocks within or near to the Gnangara pine plantation system over 30 days from 2 February to 1 May 2009. We used groups (i.e. flocks) as the sampling unit for behavioural observations rather than individuals because non-roosting flocks are highly mobile and birds do not have individually-distinctive markings allowing individuals to be monitored over time. To locate flocks, we surveyed the study area by vehicle, working either from before sunrise until midday or from midday until sunset. Our aim was to locate flocks associated with the plantations in order to undertake behavioural observations. We chose not to follow a follow a systematic survey route or to stratify survey effort by habitat type because the size and layout of the plantation system made it impractical to assess spatial distribution using a vehicle-based survey (cf the use of feeding trace). When birds were observed, we conducted a 5–10 minute ‘behavioural survey’ to collect location, behavioural (e.g. activity state), abundance, and habitat data. We classified flock activity according to nine pre-determined activity states: Roost-Overnight (over-night roosting); Roost-Rest (long-term roosting: >1 hour); Roost-Short (short duration roosting, e.g. brief roosting periods during feeding/foraging bouts or during pauses in flight); Fly (in flight); Feed (consuming or processing food items, or actively searching for food items); Preen (preening self or another individual); Feed Juvenile (Feeding juvenile/dependent chick); Drink (drinking); and Social (interaction with another individual, other than preening or feeding a juvenile). Roost-Short included situations where birds roost for short periods while travelling between distant points (e.g. between roost sites and feeding areas) and while feeding (e.g. pausing during a feeding bout to observe or listen).

If observational conditions allowed, we initiated a ‘flock follow’ using a point-sampling protocol following the completion of the behavioural survey. During flock follows, we collected these data at 15 minute intervals: numbers of birds present (actual or estimated), food plants consumed, habitat type(s) used, and activity state(s). The point samples were based on instantaneous scan sampling of the flock under observation, in which the observer assessed the activity state and habitat use of many individuals as possible [31], [32]. If multiple activity states were observed, or if flocks were using two or more habitat types, the observer counted the numbers of individuals engaged in each activity state or present in each habitat type and used these values to determine the proportions of individuals for each activity or habitat type [31], [32]. Scan samples were conducted so as to avoid bias toward any sex or age class and to ensure that less visible birds (i.e. birds within vegetation or on the ground) were observed. Observations were only classified as flock follows if flocks were observed for at least 15 minutes and thus included at least two point samples (i.e. the initial point sample and one additional point sample).

Behavioural observations were conducted over 30 days between 2 February and 1 May 2009 and included 76 sightings of Carnaby’s Cockatoos and 40 flock follows. We conducted flock follow observations for 97.3 hours (mean follow duration: 2.3 hours; median range: 0.25 to 7.4 hours) and completed 419 point samples. For the flock follow study period, mean maximum daily temperatures in the Perth metro area were: February 31.8°C, March 29.4°C, and April 27.6°C (BOM, 2009). Day-length (sunrise-sunset) varied from 13.6 to 10.9 hours.

### Activity patterns

To quantify activity patterns, we determined the predominant activity state of flocks for each point sample, as well as recording any additional (or supplementary) activity states. The predominant activity state of a flock was defined as the activity state of ≥50% of individuals during the scan sample [31], [32]. Supplementary activity states were defined as the activity state of <50% of individuals observed in a point sample.

We present the proportions for each predominant activity state (PDA): (a) relative to the total number of point samples (no. of point samples for PDA /total number of point samples) and (b) standardised to a 12-hour day. Standardisation to a 12-hour day was achieved by grouping 15-minute point samples within six one-hour intervals from (a) sunrise to 5∶59 after sunrise and (b) sunset to 5∶59 before sunset. Activity budgets for the 12-hour day are the mean values of the proportions for each PDA during the 12 one-hour sampling intervals. Similar proportions were obtained for time budgets based on all point samples and the subset of point samples standardised to a 12-hour day.

### Food plants

To obtain information on the food plants consumed by Carnaby’s Cockatoos, we recorded all food plants being consumed by birds at each point sample. In some situations it was not possible to identify the food item to species. As our interest was in identifying the food plants used and their relative frequency of use across point samples, we recorded any instance of a food plant being eaten at a point sample and did not attempt to account for the number of birds feeding on a particular food plant at a point sample. It was not uncommon for the majority of a flock to be feeding in one habitat (e.g. a pine plantation) and a small number of birds to be feeding on another food source in an adjacent habitat (e.g. *Banksia* woodland). Birds also sometimes switched between food sources within the same foraging bout, e.g. feeding on pine for a period of time and then feeding on other food sources, such as *Banksia* and *Hakea* species. Thus, the food plant data is best understood as indicating the range of food types consumed and approximate frequencies of consumption for birds observed during flock follows.

### Use of habitats for feeding and roosting

To investigate how birds used pine plantations and habitats in the surrounding landscape for feeding and roosting, we recorded the habitats used during flock follows. The predominant habitat type of a flock was defined as the habitat type occupied by ≥50% of individuals during the scan sample. Supplementary habitat types were also recorded and were defined as the habitat type occupied by <50% of individuals observed in a point sample. We defined four anthropogenic habitat types based on the dominant vegetation and land use: *Pine Plantation* (stands of *Pinus* spp. of any age or condition); *Homestead* (suburban or semi-rural properties such as households and farms); *Market Gardens* (agricultural/horticultural areas with a standing or fallow crop of fruits, nuts, fallen seed, or other man-made food source); and *Modified Land* (human-modified landscapes not fitting other habitat type descriptions, e.g. quarries). We defined four native vegetation habitat types: Banksia Woodland (woodland of *Banksia* spp., typically *B. attenuata* or *B. menziesii*), Tuart Woodland (woodland of *E. gomphocephala*); Other Woodland (woodland consisting of canopy-forming species other than *Banksia* spp. or *E. gomphocephala*); and Dryandra Thicket (shrubland of *B. sessilis*).

### Statistical Analyses

Significant differences (P<0.05) between months in monthly mean abundances of Carnaby’s Cockatoos were tested by one-way analysis of variance (ANOVA) of log transformed data (SPSS 19) since the data were not normally distributed. The significance of the age of the pine plantations and the geographic location of the 3 pine blocks (Gnangara, Pinjar and Yanchep) in determining the patterns of usage were each tested by one-way analysis of variance (ANOVA) since the variable pine ages in the different geographic locations led to an unbalanced design which precluded a two-way ANOVA. Cone mass and number of cones eaten were correlation tested (Pearson’s correlation calculated for the period from August 2009 to June 2010). The spatial distribution of the percentage of cones used across the plantations was interpolated from the 61 samples sites using the 3D graphing option in Grapher 9 (Golden Software, CO, USA).

## Results

### Pine availability, energetic and nutritional qualities

**Figure 2:**
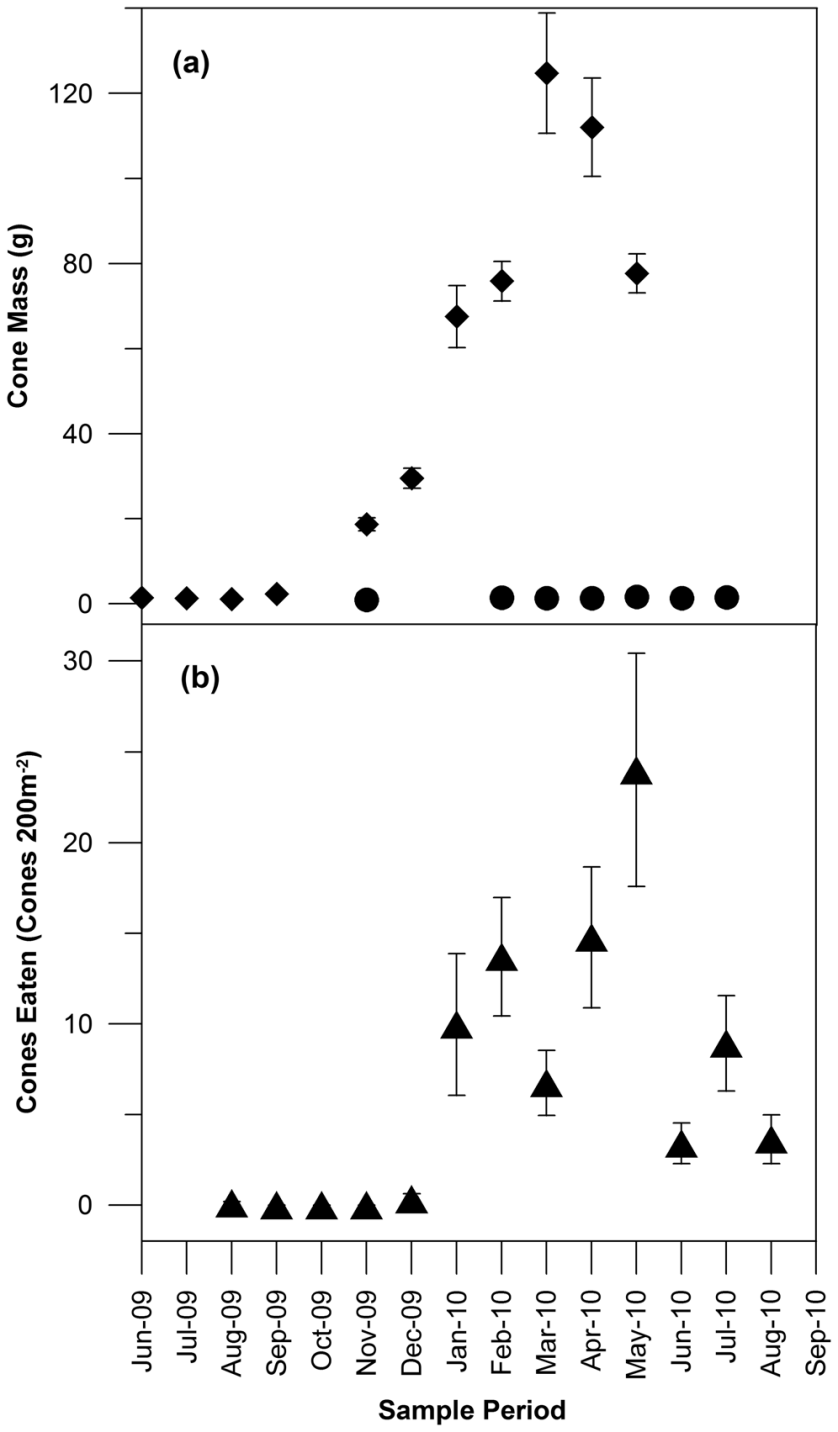
Seasonal growth (a) of two age cohorts of pine cones (pollinated in 2008 to mature in 2010–solid diamonds and pollinated in 2009 to mature in 2011–solid circles) and the seasonal use of pine cones (b) by Carnaby’s Cockatoos at 9 sites (mean±SE of 3 transects of 200 m^−2^ at each site) across the pine plantations of the Gnangara.

The mass of the cones pollinated in 2008 increased rapidly from November 2009 until March 2010 with cones beginning to mature (assessed visually as the point when the cones begin to dry out and change from green to brown in colour) by January 2010 (Figure 2a). Cones pollinated in 2009 remained constant in size for the whole monitoring period. The number of seeds in the mature cones was 129±4.54 (Mean ± SE, n = 62) thus providing an average density of 158 025 seeds ha^−1^.

The chemical compositions of the seeds of the native and non-native plants eaten by Carnaby’s Cockatoos were typical of nuts [26], [28] in terms of the energy available per unit of mass which averages 16 864 J g^−1^ across all the species (Table 1) with the pines being about 27% higher in energy than the native species. Nitrogen was more variable with an average of 9.0% across all species and the pines had nearly 45% less protein than the high N seeds of the Proteaceae (*Banksia* and *Hakea* spp.) Protein in pine seeds is more similar to other native species of the Myrtaceae (*Eucalyptus* and *Corymbia* spp.) and Fabaceae (*Acacia* spp.). The large differences among species, in both seed size and the ratio of cotyledon mass to seed testa, leads to a 16 fold difference in the number of seeds of each species that are required to meet the daily minimum field metabolic requirements of the birds (FMR given by Cooper et al. [29]). The energetic contributions *Banksia* spp. and *Corymbia calophylla* can make to Carnaby’s Cockatoo diets are clearly evident (Table 1), with pine being less rewarding at the individual seed level. However, these estimates of food energy availability do not include estimates of energy expended in foraging for food and the handling times associated with extracting the seeds from the various species.

**Table 1:** Seed mass, cotyledon percentage of seed mass, energy, fat, nitrogen and protein contents of 12 native and three non-native (#) Carnaby’s Cockatoo food plants.

Species	Seed Mass (g)	Cotyledon Mass (% Seed Mass)	Energy (J g^−1^)	Fat (%)	N (%)	ND Fibre (%)	Crude Protein (%)	Seeds to Meet FMR (726 kJ d^−1^)
*Acacia saligna*	0.0185	56	18857	15.0	5.1	12.7	27.0	4030
*Banksia attenuata*	0.1081	74	17267	8.4	14.1	25.7	74.7	567
*Banksia grandis*	0.0764	81	18471	11.0	14.6	8.1	77.4	660
*Banksia littoralis*	0.0214	73	17583	8.9	14.6	13.3	77.4	2814
*Banksia nobilis*	0.0188	76	18381	12.8	13.8	13.1	73.1	2933
Banksia sessilis^*^	0.0068	78	18503	12.6	12.0	11.3	63.6	6109
Brassica napus^*^ ^#^	0.0042	81	21769	39.1	3.6	33.9	19.1	9108
*Corymbia calophylla*	0.0985	59	19137	26.3	7.1	34.7	37.6	764
Eucalyptus marginata^*^	0.0149	68	11990	5.1	2.0	67.0	10.6	7546
*Hakea incrassata*	0.0553	81	17146	15.4	10.4	22.6	55.1	1002
*Hakea laurina*	0.0222	75	20248	25.6	10.1	20.8	53.5	2346
*Hakea preisii*	0.0137	46	19044	17.6	9.9	16.4	52.5	6547
*Pinus pinaster* ^#^	0.0505	40	24922	41.5	7.4	4.2	39.2	1471
*Pinus pinea* ^#^	0.4774	9	21334	32.2	6.3	23.4	33.4	873
Xanthorrhoea preissii^*^	0.0271	56	16799	22.8	4.4	52.9	23.3	2133

The final column shows the predicted number of seeds (corrected for testa mass) required by a Carnaby’s Cockatoo to meet its daily minimum field metabolic rate (FMR = 726 kJ d^−1^) as reported by Cooper *et al*. [29]. ^*^Not dehusked prior to analysis.

### Carnaby’s Cockatoo abundances

**Figure 3:**
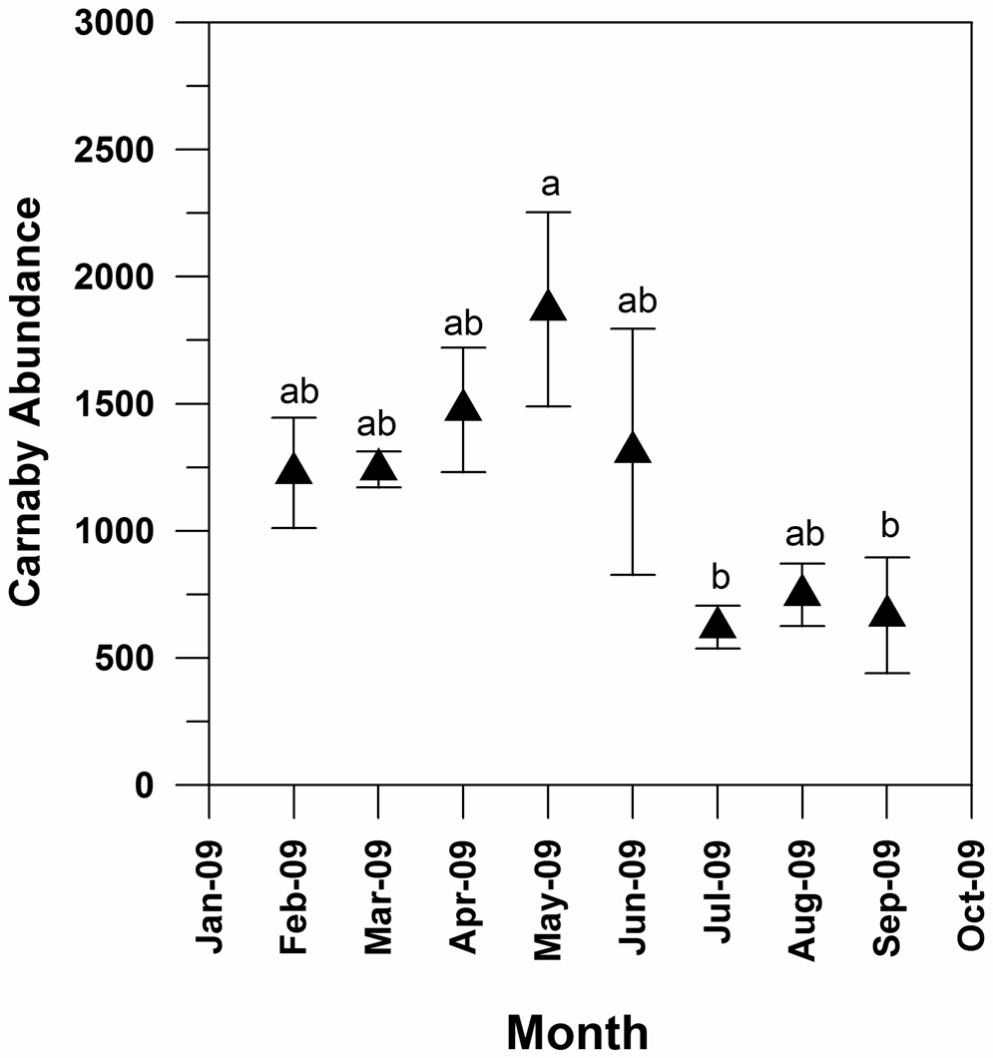
Number (monthly mean ± SE) of Carnaby’s Cockatoos roosting at sites (n = 14) within or adjacent to the *Pinus pinaster* plantations of the Gnangara. Different letters indicate Tukey post-hoc significant differences (P<0.05) between months after One-way ANOVA of log transformed data: F_7,20_ = 4.426, p<0.05).

The number of Carnaby’s Cockatoos counted at the 14 roost sites differed significantly between months with the highest abundances in May and the lowest in September (Tukey post-hoc test after One-way ANOVA of log transformed data: F_7,20_ = 4.426, P<0.05). Carnaby’s Cockatoos were present in high abundances from February to June 2009 with the mean weekly roost counts of c. 1200 individuals per week in each month (Figure 3). However, the weekly counts were extremely variable. For example, both the maximum (2588) and minimum (415) number of birds counted was recorded three weeks apart in April and May. Individual roost site counts were also extremely variable with some sites recording no birds in one week followed by more than 1000 birds the next. After June, the number of birds declined with the weekly average reduced to 800 birds.

### Feeding activity within pine plantations

**Figure 4:**
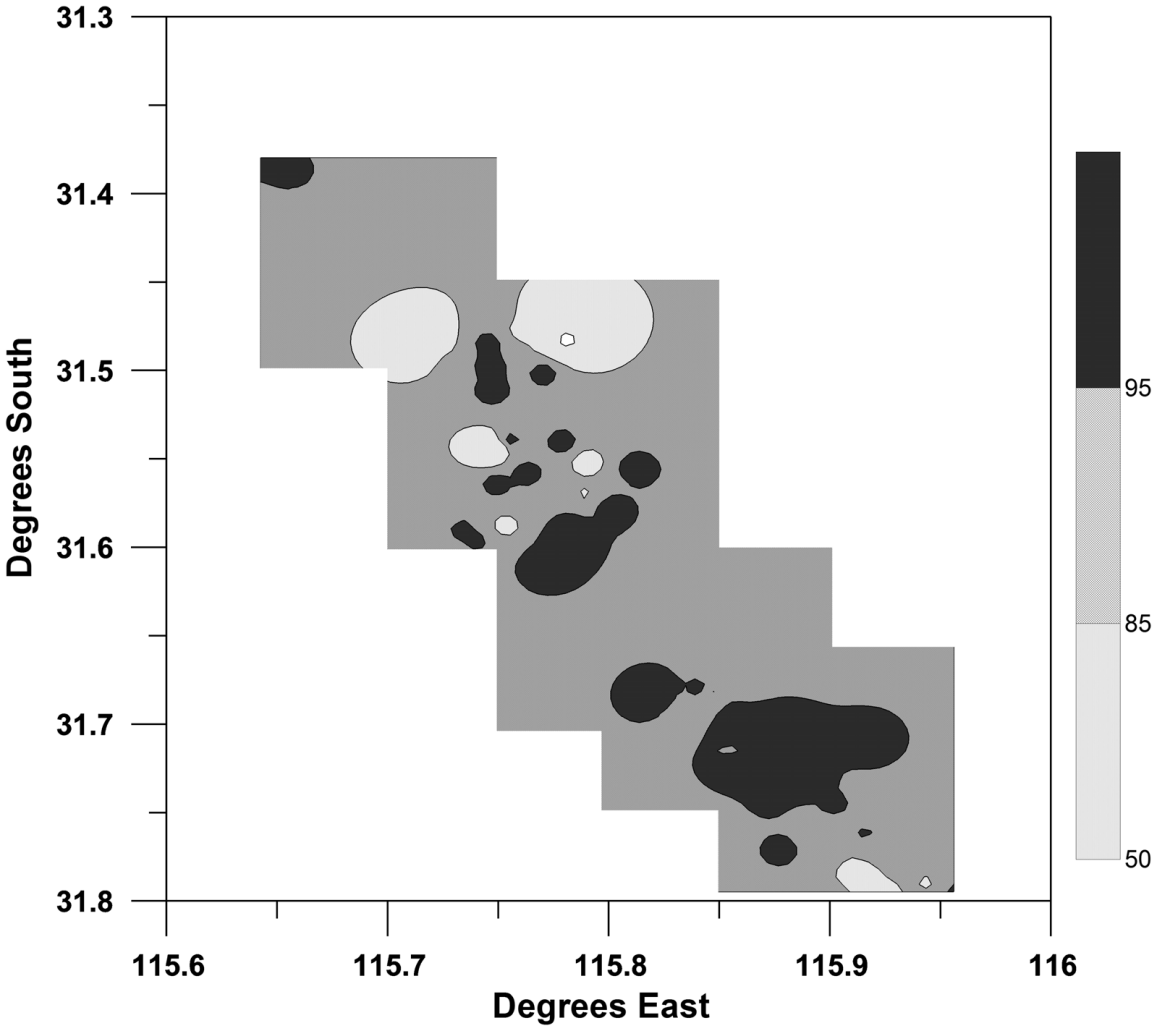
Interpolated spatial pattern of pine cone use (% of total available) by Carnaby’s Black Cockatoo across the pine plantations of the Gnangara. Shaded areas indicate categories where available pine cone use is>95%, 85–94%, 50–84% and <50%.

**Figure 5:**
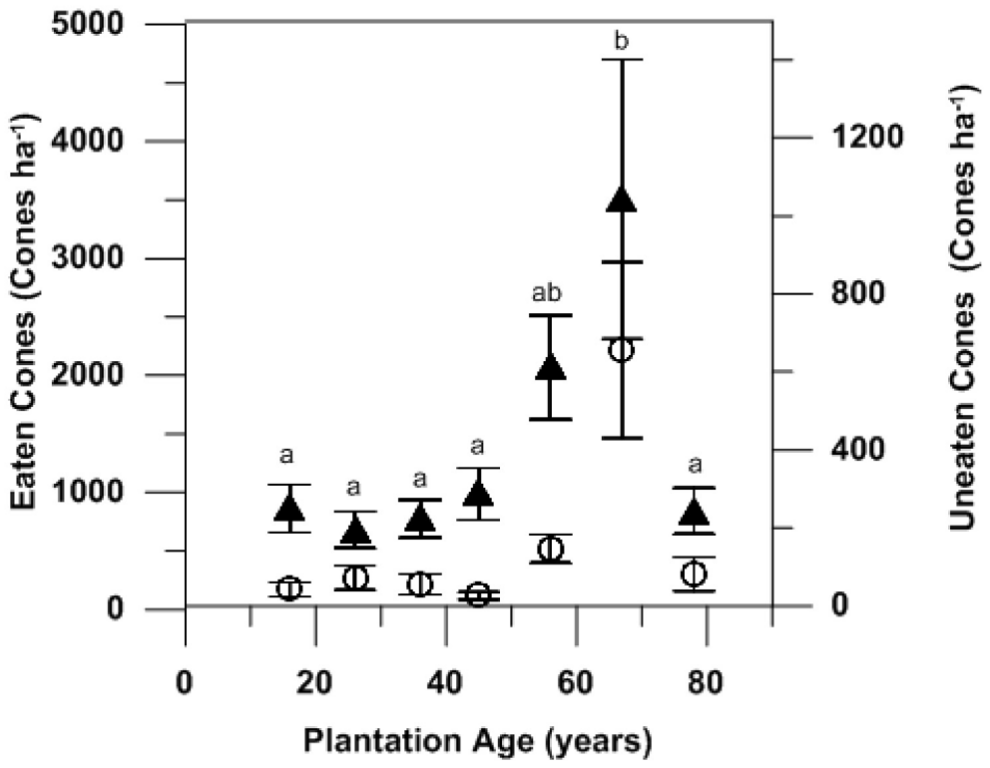
Numbers of eaten (fully or partially-solid diamond) and uneaten (retained in canopy–open circle) pine cones sampled across 122 quadrats at 61 sites across the Gnangara pine plantations in July to August 2009. Different letters indicate Tukey post-hoc significant differences (P<0.05) between plantation ages after One-way ANOVA F_6,115_ = 5.499, p<0.05).

Nearly all (121 of 122 plots) sampling plots contained feeding traces and all sections of the Gnangara plantations showed high cone use (>85% of available cones eaten, Figure 4). The one plot that did not contain any feeding trace had a very low cone density (<50 cones ha^−1^). Feeding traces were significant higher (Tukey post-hoc test after One-way ANOVA F6_,115_ = 5.499, P<0.05) in pine stands of 60–70 years (with the mean number of eaten cones exceeding 3000 cones per ha (Figure 5). Pines stands of 18 to 50 years in age also had a high incidence of feeding trace (600–1000 eaten cones ha^−1^). The mean density of feeding trace was 1225 cones ha^−1^ across all sites and there were no differences between pine stands in the three geographically distinct blocks (Gnangara, Pinjar and Yanchep) making up the total plantation area (One-way ANOVA F_2,61_ = 0.59, P>0.05).

The seasonal pattern of feeding on pine is correlated with cone mass (Pearson coefficient = 0.68, p<0.05) and the birds appear to exploit the cones as early after seed maturation as possible (Figure 2b). Feeding traces showed a strong seasonal pattern with no feeding before January 2010 thereafter reaching a peak of activity in April to May of 2010 (Figure 2b) when almost all cones had matured. Feeding dropped off from June 2010 presumably because the pine cone resource had been fully exploited.

### Daily activity patterns

**Figure 6:**
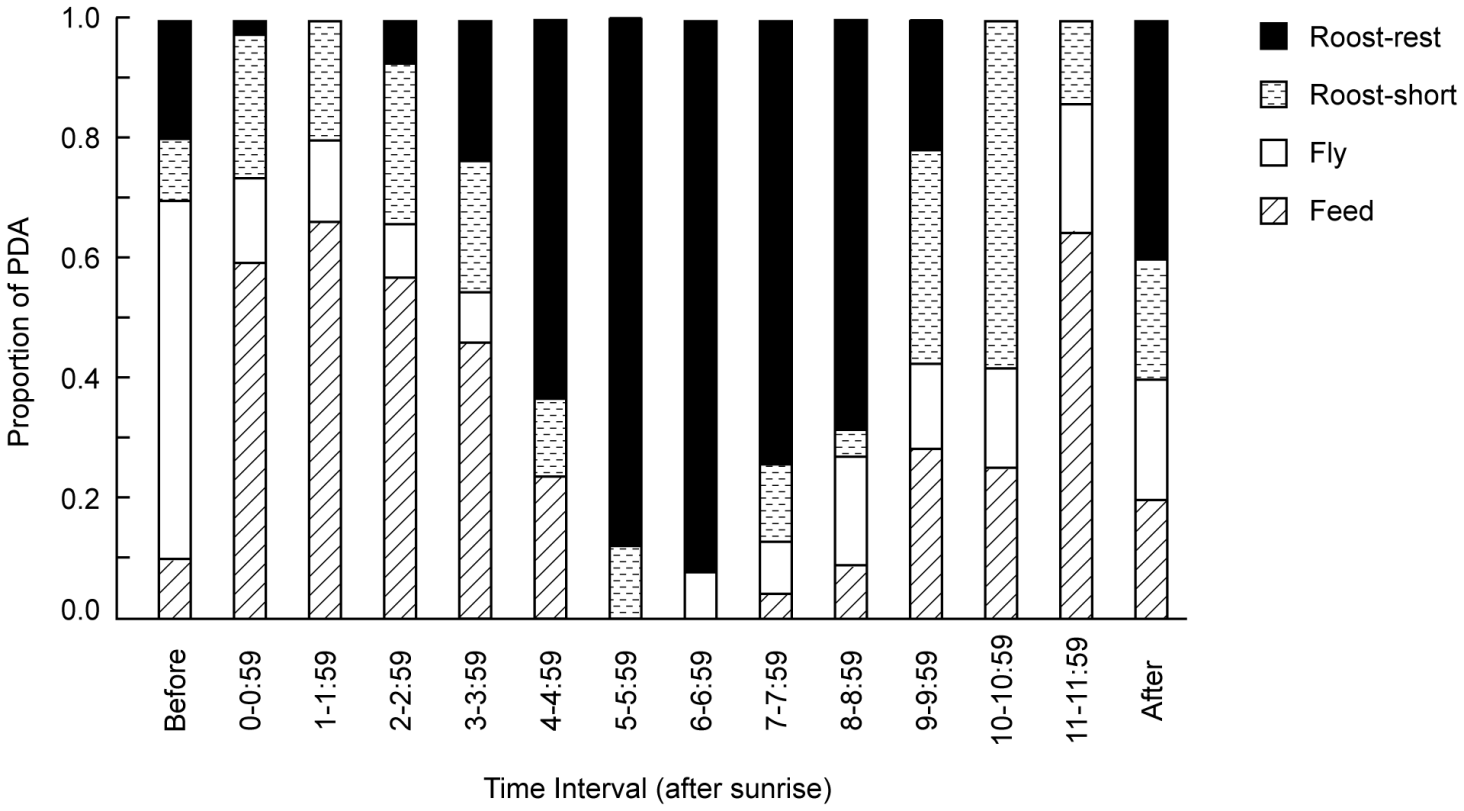
Distribution of predominant activity (PDA) states for Carnaby’s cockatoos from before sunrise to after sunset standardised to a 12-hour day.

Flocks were generally either resting at roost or feeding (Table 2). The rest of their daily activity was devoted to some kind of transitional movement, either between roost sites and other habitats (e.g. drinking sites, feeding areas) or between different feeding patches, with birds in flight or roosting for short periods of time. Flocks exhibited a distinct bi-modal activity pattern characterised by active periods after sunrise and before sunset and a long rest interval at midday. After roosting over-night, they became active just before dawn and moved away from roost sites, sometimes after feeding briefly close to the roost site. Upon leaving roost sites, flocks were typically active and predominantly feeding (or travelling) for 3–4 hours after dawn, then remained at Roost-Rest for several hours during the middle of the day, before becoming active again (and largely feeding) about 3–4 hours before sunset (Figure 6).

**Table 2:** Time budget for predominant activity (PDA) state of Carnaby’s Cockatoos standardised for a 12-hour day (percentages shown in parentheses).

Predominant Activity (PDA)	Number of point samples (%) (*n* = 419 point samples)	Hours of 12-hour day (%) (*n* = 376 point samples)
Roost-Rest	156 (37.2%)	4.4 (36.7%)
Feed	145 (34.6%)	3.9 (32.2%)
Roost-Short	75 (17.9%)	2.4 (19.9%)
Fly	42 (10.0%)	1.3 (10.9%)
Drink	1 (0.2%)	<0.1 (0.3 %)

Feeding was a frequent supplementary activity (n = 85 point samples), meaning that some feeding occurred in the majority of point samples (n = 230 of 419 point samples, 54.9%). Feeding occurred as a supplementary activity during active periods when the predominant activity was Roost-Short and during inactive midday periods when the predominant activity was Roost-Rest. Small numbers of birds fed during Roost-Rest periods by ground-feeding on fallen pine cones or shrubs and on seeds in a fallow market garden and by canopy-feeding on pine cones.

Four activity states were never or only rarely recorded as the PDA during point samples: Preen, Feed Juvenile, Social, and Drink. Preening and feeding of juveniles were associated the midday roosting period. Social interaction occurred throughout the day and during both feeding bouts and roosting periods. Interactions included antagonistic ‘squabbles’ between individuals and male displays to females.

We observed 14 drinking events. These occurred during the morning immediately after leaving the overnight-roost (n = 3), during the active morning period (n = 6), just before the midday roost (n = 3), and in the evening just before flocks assumed the overnight roost (n = 2). Drinking bouts often lasted for only a few minutes, with most birds at drink sites roosting in nearby trees rather than drinking.

### Food plants

Carnaby’s Cockatoos fed on native and non-native food plants, including *Pinus* spp., three *Banksia* species (*B. sessilis*, *B. attenuata*, *B. prionetes*), several *Hakea* spp., Marri (*Corymbia callophylla*), insect larvae, market garden crops and fallen seed, orchard fruit or nut (species undetermined), and unknown food sources on the ground (Table 3). Birds were most frequently observed consuming pine (n = 103 of 230 point of Feed point samples), *Banksia* spp. (not including *B. sessilis*) (*n* = 46 point samples), vegetation and seed in market gardens (n = 42 point samples), and *B. sessilis* (n = 41 point samples) (Table 3). Other food items were fed on in less than 10% of point samples. Ground foraging occurred in more than half of all point samples in which feeding was observed (n = 123 point samples).

**Table 3:** Food items consumed by Carnaby’s Cockatoos during point samples in which feeding activity was recorded, either as the predominant activity (PDA) (*n* = 145) or as a supplementary activity (*n* = 85) (*n* = 230 total point samples with percentages in parentheses).

Food item	No. of point samples in which food item eaten
Pine cone (canopy)	81 (35.2%)
Pine cone (ground)	61 (26.5%)
**Pine** (total)	103 (44.8%)
Market Garden (ground vegetation or seed)	8 (3.5%)
Market Garden (ground seed-fallow field)	34 (14.8%)
**Market Garden** (total)	42 (18.3%)
*Banksia* spp.* (cone or flower spike-canopy)	43 (18.7%)
*Banksia* spp.* (cone-ground)	8 (3.5%)
***Banksia*** spp.* (total)	46 (20.0%)
Dryandra (*Banksia sessilis*, seed)	41 (17.8%)
*Hakea* spp. (seed or flower)	11 (4.8%)
Grubbing (observed/probable)	14 (6.1%)
Marri (flower, nectar, insect)	7 (3.0%)
Horticultural fruit/nut (canopy/ground)	3 (1.3%)
Unknown food item (ground)	12 (5.2%)

* - not including *Banksia sessilis*.

### Use of habitats for feeding and roosting

Flocks used both anthropogenic habitats and native vegetation with feeding observed in eight habitats and roosting or resting in five habitats (Table 4). Birds used pine plantations for feeding as well as for midday and over-night roosting. Most habitats in the landscape around the pine plantations were used either for feeding (Market Garden, Banksia Woodland, Dryandra Thicket) or for roosting (Homestead, Tuart Woodland, Other Woodland) depending on the mix of available food plants and roost trees in each habitat type.

**Table 4:** Observed locations of predominant activities (Feed and Roost-Rest point samples) by predominant habitat type.

Habitat Type	No. of point samples (%)	
	*PDA: Feed* (*n* = 145)	*PDA: Roost-Rest* (*n* = 156)
Pine plantation	66	55*
Market garden	36	0
Homestead	5	41
Modified land	1	0
*Banksia* woodland	12	3
Tuart woodland	3	36
Other woodland	4	21
*Dryandra* thicket	18	0

* - includes 2 Roost-Overnight point samples taken before sunrise.

## Discussion

The results of our study illustrate the strong ecological association between Carnaby’s Cockatoos and plantation pine. The broad spatial extent of cockatoo feeding activity within the Gnangara pine plantation system; the near-complete removal of the system’s annual standing crop of pine cones; the close coincidence of cone maturation with the onset of feeding activity; the overlap between cone availability and high Carnaby’s Cockatoo abundances; the energetic and nutritional value of pine seed as a food source; and the use of pine stands for both feeding and roosting all indicate this strong ecological association of Cananby’s Cockatoos and pines. In addition the activity pattern of Carnaby’s Cockatoos, associated with plantations, also appears closely aligned with the unique features of the plantation landscape including an easy access to water and the provision of an abundance of midday roosting habitat.

The Gnangara plantation system represents the largest land use entity within the Perth metropolitan area. At the time of this study, it encompassed 15 000 ha and extended for more than 45 km along its north-south axis. Carnaby’s Cockatoos fed throughout the plantation system and, appear to deplete the annual standing crop of pine cones, confirming observations made in earlier studies [12], [14]. Carnaby’s Cockatoos consumed more than 85% of annual cone production overall, with removal rates in some areas exceeding 95%. Removal rates were highest in the 50–70 year old stands, although some feeding occurred in stands of all age cohorts. The peak in feeding activity within 50–70 year old stands likely reflected the effect of stand thinning. Stand thinning allows the trees remaining in a stand to develop larger and fuller canopies and to sustain high rates of cone production. In contrast, younger stands with higher stem densities experience greater competition for resources (light, water and nutrients) and therefore reduced cone production and seed set [33]. *Pinus pinaster* matures (i.e. produces seed) at about five years of age [34] and our results are consistent with the observations by Perry [12] that birds in the Gnangara feed on all trees that produce cones.

The presence and abundance of feeding trace mirrored the pattern of cone maturation. Feeding activity (i.e. feeding trace) within the plantation system was negligible when cones were not present or were immature (August and December 2009), but increased rapidly once cones matured in January and continued at a high level until June 2010. This period also coincided with the highest mean roost counts. Feeding activity was most intense during the non-breeding season, which is consistent with observations recorded in previous studies [10], [35], [36]. Finn and et al. [8] estimated that c. 3000–4000 birds were associated with the Gnangara pine plantations system during the non-breeding period. The distinctiveness of this seasonal pattern of feeding activity suggests that flocks time their use of the plantation system to coincide with pine seed maturation. The summer to autumn maximum in pine seed availability is also a crucial phase in the breeding cycle as mated pairs have fledgling chicks that are dependent on them for food [37], [38]. Rearing chicks in an environment with easily available food resources, such as pine, could be critical for maintaining populations in landscapes that are highly fragmented and which continue to change through clearing or decline of native vegetation [39], [40].

The roost counts, though variable, are consistent with previous studies of roosting abundance within the Gnangara area. Saunders [10] reported that the number of Carnaby’s Cockatoos observed drinking at the western margin of the Gnangara plantation system was very low from July through December but increased rapidly in January and peaked in March. Variability in roost counts may reflect, among other things, responses to the availability of local food sources. Finn et al. [8], for example, reported large (e.g. 1000+ individuals) flocks feeding on *Banksia sessilis* seeds in late April and early May. These observations coincided with low or absent counts at many of the monitored roost sites, indicating the movement of flocks within the study area. Thus, the roost counts are best seen as providing an index of the relative abundance of birds associated with pine plantations rather than a measure of absolute abundance. Additional behavioural observations are required to interpret trends in abundance and the usage of specific roost sites.

Although the exact factors influencing food selection in the Perth region during the non-breeding season are not clear, pine seeds have several characteristics that enhance their suitability. Firstly, the seeds are available (i.e. mature cones are present) during the non-breeding season. Secondly, the seeds are easily extracted from the cones [8], [14]. Observations of Carnaby’s Cockatoos feeding on *P. pinaster* cones indicate that birds can consume all of the seeds from a single cone in less than 10 minutes (W. Stock and H. Finn, personal observation). Thirdly, the seeds are likely to be highly palatable to the birds because of their nutritional value [41], [42]. Finally, the high fat content means that pine seeds have a high energetic value, a trait that is further enhanced by the large number of seeds per cone and per ha of plantation.

The energetic and protein contents of pine seed and seed from native food plants are typical of those found in nuts. The energy content of all food sources examined was broadly similar, with *P. pinaster* seeds having higher energy contents than any of the native myrtaceous and proteaceous species. The protein content of pine seeds (38%) was much less than for native *Banksia* (62–76%) and *Hakea* spp. (52–54%). To our knowledge, the daily protein requirements of Carnaby’s Cockatoos have not been determined, making it difficult to determine the implications of the lower protein content in pine. Protein requirements for avian granivores generally increase with body size [42]. Smaller psittacines, such as cockatiels and budgerigars, have been reported to have maintenance food protein requirements of 11% or less [42], although requirements increase when birds are raising chicks (e.g. 20% in breeding cockatiels [43]). These values suggest that the protein content of *P. pinaster* seed is well above levels likely to be limiting for maintenance and growth in Carnaby’s Cockatoos. Plant seeds vary considerably in their amino acid composition, making it important to consider the quality of the protein source (essential versus non-essential amino acids), particularly where the protein quality of a plant may lead to a limitation of one or more amino acids [44]. However, the amino acid profiles of *Banksia* and pine seed proteins are very similar (both being dominated by arginine, asparagine, glutamine, and alanine) [45], [46], [47], indicating the protein quality is probably not a consideration for these foods.

Nutritional characteristics probably do not influence the selection of seed-based food sources as much as other factors, such as the morphological traits of the seed and cone or capsule (e.g. ease of processing); the energy density of a food plant (e.g. the number of seeds per cone or capsule); and tree or landscape determinants of food availability (e.g. stand density, number of cones per tree) [25], [48]. Feeding trials with captive birds may offer opportunities to test hypotheses about seed preference [49]. Feeding on pine appears to allow birds to easily meet their daily energetic requirements. Cooper et al. [29] studied the metabolic rates of captive Carnaby’s Cockatoos and estimated a field metabolic rate of 726 kJ d^−1^. They also measured the energetic content of *P. radiata*, which is widely used in plantation systems south of Perth [40]. Based on these measurements and counts of the number of seeds per pine cone, Cooper et al. [29] calculated that 18 *P. radiata* cones would be sufficient to meet daily metabolic requirements for Carnaby’s Cockatoos. Applying the same approach to our estimates for the energetic content of *P. pinaster*, we estimate that only 11 *P. pinaster* cones are needed to meet daily metabolic requirements of Carnaby’s Cockatoos and 15 000 ha of pine would thus be able to support the energy needs of 9280 birds for a period of six months. A 10-minute cone processing time suggests that, leaving other factors aside, Carnaby’s Cockatoos could meet their daily metabolic requirements by feeding on *P. pinaster* for less than two hours per day. Thus, from an energetic perspective, pine is analogous to other anthropogenic food sources having a high energetic return thereby allowing birds to forage efficiently and to obtain the necessary food with little time invested.

The Gnangara landscape allows provides two additional ecological benefits for Carnaby’s Cockatoos: a reliable water sources during summer and suitable roosting habitat near to feeding habitat. Maintaining hydration is a key ecological challenge for black cockatoos, particularly in summer [1], [37], [50], [51]. Large Psittacines must drink at least once a day during high temperatures and must carefully manage their daily hydration status in order to maintain normovaelemia and blood-electrolyte balances [52], [53]. A recent mortality event emphasised the vulnerability of Carnaby’s Cockatoos to heat stress. In January 2010, 208 birds died in the Hopetoun and Munglinup region in southern Western Australia when air temperatures reached 47°C combined with a 60 km h^−1^ northerly wind [54]. Post-mortem examination attributed the deaths to heat stress and subsequent dehydration, as no potential toxins were detected and birds had recently ingested food [54]. A midday roost period allows Carnaby’s Cockatoos to avoid heat stress when temperatures and solar radiation are most intense [37]. As the pine stands provide a suitable habitat for the midday roost, flocks are able to remain at feeding sites after the morning feeding bout, thereby avoiding energy expenditure and flight activity during peak temperatures. Similarly, the presence of abundant natural and artificial water sources along the margins of the plantations allows easy access to water when birds are leaving or returning to their over-night roosts.

Land managers have long been aware of the strong ecological association between Carnaby’s Cockatoos and pine plantations. Perry [12] was the first to document a behavioural innovation which now constitutes an important (and enduring) feature of the “modern” ecology of Carnaby’s Cockatoos–the species’ annual visitations to pine plantations to feed on an abundant and energy-rich (yet decidedly anthropogenic) food. This species has become habituated to the availability of this food source since the first pine trees planted in the Perth area began to bear mature cones in the 1930s [12]. The ecological patterns described by Perry are still largely consistent with our contemporary observations, as well as those of Saunders in the early 1970s [14]. Thus, plantation pine has supported populations of Carnaby’s Cockatoos in a predictable manner for more than 75 years, a period in which the total population size of the species has declined by more than one-half and its range contracted by more than one-third [4], [39]. The hypothesis that the availability of such an abundant and energy-rich food source has allowed for Carnaby’s Cockatoo abundances well in excess of those recorded before the pine was planted is intuitively appealing, but unfortunately there are currently no comparative data describing the carrying capacity of native *Banksia* woodland for Carnaby’s Cockatoos.

The strong ecological association between Carnaby’s Cockatoos and pines seen in this study clearly indicates that the establishment of broad-scale pine plantations in the 1940s inadvertently created a situation in which an endangered species has become reliant upon a non-native introduced food species. The decision to harvest all remaining pine stands without replacement means that this food resource will be removed by 2031 or earlier [6], [22]. Though the consequences of this change in land use for Carnaby’s Cockatoos have been considered [8], [17], [22], it is highly unlikely that the current plans for the re-establishment of native vegetation will provide for anything near the abundance of food the pine stands now provide. The harvesting of pine plantations will cause birds to make greater use of other food sources in the Perth and surrounding areas, which is likely leading to greater conflict with certain land users (e.g. market gardeners, nut growers and horticulturists). It is also evident that as the pine plantations are harvested, there is an urgent need to establish alternative food sources if the Carnaby’s Cockatoo populations currently using the area are to be retained and risks to the survival of the species avoided.
